# Preparation and Characterization of Polyelectrolyte Complexes of* Hibiscus esculentus* (Okra) Gum and Chitosan

**DOI:** 10.1155/2018/4856287

**Published:** 2018-04-24

**Authors:** Vivekjot Brar, Gurpreet Kaur

**Affiliations:** Department of Pharmaceutical Sciences and Drug Research, Punjabi University, Patiala, Punjab 147002, India

## Abstract

Polyelectrolyte complexes (PECs) of Okra gum (OKG) extracted from fruits of* Hibiscus esculentus* (Malvaceae) and chitosan (CH) were prepared using ionic gelation technique. The PECs were insoluble and maximum yield was obtained at weight ratio of 7 : 3. The supernatant obtained after extracting PECs was clearly representing complete conversion of polysaccharides into PECs. Complexation was also evaluated by measuring the viscosity of supernatant after precipitation of PECs. The dried PECs were characterized using FTIR, DSC, zeta potential, water uptake, and SEM studies. Thermal analysis of PECs prepared at all ratios (10 : 90, 20 : 80, 30 : 70, 40 : 60, 50 : 50, 60 : 40, 70 : 30, 80 : 20, and 90 : 10; OKG : CH) depicted an endothermic peak at approximately 240°C representing cleavage of electrostatic bond between OKG and CH. The optimized ratio (7 : 3) exhibited a zeta potential of −0.434 mV and displayed a porous structure in SEM analysis. These OKG-CH PECs can be further employed as promising carrier for drug delivery.

## 1. Introduction

Chitosan (CH) is among the most commonly employed natural polymers for drug delivery systems due to its biocompatible and biodegradable nature. It is a naturally occurring cationic polysaccharide consisting of glucosamine and N-acetyl-glucosamine obtained by partial deacetylation of chitin [1]. It is degraded in vertebrates by lysozyme and by bacterial enzymes in the colon [2]. These properties of CH make it desirable candidate for use as excipient in drug formulations. However, high solubility of CH in acidic conditions limits its use in sustained release oral preparations. The solubility of CH is attributed to the protonation of the free amine group. If this amine group is not free then there might be a possibility of using this biodegradable polymer in oral sustained release drug delivery systems. A large number of polyelectrolyte complexes (PECs) have been reported in the literature for controlling the release of drugs. Chitosan is cationic in nature, this property can be employed in the formation of PECs using ionic cross linking with poly anions like tripolyphosphate (TPP) [3], other anionic natural polysaccharides such as alginate [4, 5], pectin [6], carrageenan [7], xanthan gum [8], and gum kondagogu [9], synthetic anionic polymers such as poly acrylic acids [10], and semisynthetic polymers such as carboxymethylcellulose [11].

Okra gum (OKG) is anionic polymer obtained from the fruits of* Hibiscus esculentus* (family Malvaceae) consisting of galactose, rhamnose, and galacturonic/glucuronic acid as monomers [12]. It is used in pharmaceutical preparations as binder [13] and film coating agent [14], in colon targeting [15], and in buccal delivery [16].

CH and OKG both cannot be used alone in the formulation of sustained release dosage forms as they are limited by their high solubility. Complexation of CH with anionic polymers has been reported to decrease its solubility. This property is useful in sustaining the drug release. The interaction of polymer with an oppositely charged polymer results in an exchange of counterions and it is accompanied with an increase in entropy [17]. This increase in entropy suggests that the complexation reaction is spontaneous. The formation and properties of PECs depend on molecular weight, density of charge, and degree of neutralization of the polymers employed [18]. The PECs between oppositely charged polymers impart characteristic final properties to polymers (solubility, swelling, and rheology). The PECs when used in drug delivery may control the release of drug from the dosage form. Also, depending on different polymeric factors and formulation conditions the PECs can be formulated as different dosage forms such as microcapsules [19], gel nanoparticles [20], nanoparticles [21], controlled release membranes [22], gels [8], inserts [23], films [24], and carriers for oral administration [25].

There are no published reports of formation of polyelectrolyte complexes of Okra gum so the research in hand was designed to prepare and characterize the polyelectrolyte complexes between OKG and CH. The carboxylic acid groups present in OKG may react with amine groups present in chitosan resulting in formation of polyelectrolyte complexes. These complexes may be further evaluated for their potential as carrier for drug delivery systems.

## 2. Material and Methods

### 2.1. Material

Chitosan (CH), degree of deacetylation > 80%, was purchased from India Sea Foods, Cochin, India. Acetic acid, acetone, barium hydroxide (Ba(OH)_2_), and zinc sulfate (ZnSO_4_) used in the study were purchased from Loba Chemie and were of analytical grade.

### 2.2. Extraction of Okra Gum

Okra gum (OKG) was extracted using method described by Kaur et al. [16]. Briefly, crushed Okra fruits were allowed to swell in water for 8–12 h. This was followed by filtration through muslin cloth and deproteinization with barium hydroxide (Ba(OH)_2_) and zinc sulfate (ZnSO_4_). The supernatant was then precipitated with acetone. The powdered gum was dried by freeze drying (Allied Frost, Delhi) and stored in air tight container.

### 2.3. Preparation of Polyelectrolyte Complexes

OKG and CH were dissolved separately in distilled water and acetate buffer (pH 5.0), respectively, producing a 1% w/v of polymer solutions. The OKG solution was then added to CH solution dropwise (10 : 90, 20 : 80, 30 : 70, 40 : 60, 50 : 50, 60 : 40, 70 : 30, 80 : 20, and 90 : 10; OKG : CH). The suspensions were stirred at room temperature for one hour. The suspension was then incubated for 24 h in shaking incubator (Remi shaking incubator CIS 24, Mumbai, India) at temperature of 37°C and the precipitates were separated by centrifugation at 10,000 rpm (6708 g) for 10 min (Remi cooling centrifuge, Mumbai, India), washed with distilled water, and freeze dried. The practical yield of the PECs was calculated employing the following equation:

(1) $$\begin{array}{c} Percent\;\;yield\left(\;\%\;\right)=\frac{W_{0}}{W_{t}}\times 100, \end{array}$$

where *W*_0_ is weight of PEC obtained and *W*_*t*_ is total weight of polymers taken.

### 2.4. Viscosity Measurements

The viscosity of the supernatants obtained after mixing OKG solutions with CH solution in different ratios was determined with Brookfield viscometer spindle S18 using small volume adapter (Brookfield viscometer LVDV1, Bruker, UK). Briefly, 7-8 mL sample was poured in adapter. The samples were equilibrated to a temperature of 25°C using a circulatory water bath for 15 minutes. Thereafter the viscosity measurements were carried at suitable rpm.

### 2.5. Fourier Transform Infrared (FTIR) Measurements

The spectral characteristics of different ratios of PECs were determined employing FT Infrared Spectrophotometer Model RZX (Perkin Elmer). The dried samples of PECs were compressed with KBr to form pellets for FT-IR measurement. Sixty-four scans were single-averaged at a resolution of 4 cm^−1^.

### 2.6. Thermal Analysis

The thermal properties of the PECs were evaluated using differential scanning calorimeter (EVO 131, SETARAM Instrumentation, France). The samples were prepared by weighing accurately 5-6 mg of sample and crimped in aluminum crucibles with a pin holed lid and heated in the range of 40–400°C at a heating rate of 10°C min^−1^ under a purge of nitrogen at rate of 30 mL/min.

### 2.7. Zeta Potential Measurements

Zeta potential measurements of different ratio of PECs were carried out using a Zetasizer (Malvern Instruments Ltd., Malvern, UK). The zeta potential measurements were performed using an aqueous dip cell in an automatic mode. Samples were diluted in triple distilled water and placed in a clear disposable zeta cell, with the cell position being adjusted.

### 2.8. Swelling Measurements

Accurately weighed amounts of PECs prepared employing different ratios of CH and OKG were compressed into pellets (6 mm, radial diameter) using a tablet compression machine. These pellets were positioned on top of a sponge. The sponge was previously soaked in hydrochloric acid buffer (0.2 M) solution of pH 1.2 and phosphate buffer pH 6.8 (0.2 M) in a Petri plate. The water uptake by the pellets was calculated by weighing the initial weight of the pellet before keeping it on hydration medium and after eight hours [23]. The swelling behavior of the PECs was calculated using the following equation:

(2) $$\begin{array}{c} P_{s}=\frac{\left(W_{s}-W_{i}\right)}{W_{i}}\times 100, \end{array}$$

where *P*_*s*_ is percent swelling, *W*_*s*_ is weight of the swollen pellet at time “*t*,” and *W*_*i*_ is initial weight of the pellet.

### 2.9. SEM Analysis

Sample of powdered PECs was sprinkled on the sample stub with double-sided tape, extra powder was removed and coated for 70 s under an argon atmosphere with gold coating, and then the powdered samples on the SEM grid were allowed to air dry for 10 min. Images were captured at different magnifications using SEM (Jeol JSM-6610 LV, USA) machine.

## 3. Results and Discussions

### 3.1. Extraction of OKG

The extraction technique used for the isolation of OKG from fresh pod of* Hibiscus esculentus* resulted in a yield of 0.5% w/w. The gum was pale green in color with a characteristic odor [16].

### 3.2. Yield of Complexes

**Figure 1:**
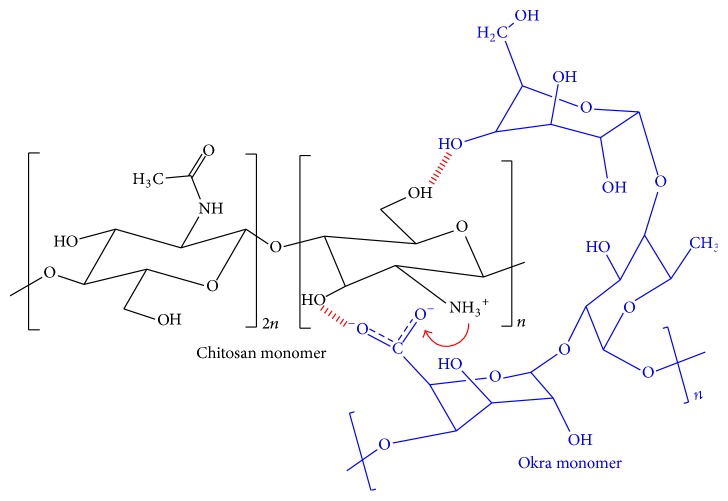
Electrostatic interactions between the monomers of OKG and CH.

The percent yield of the PECs obtained at different ratios is depicted in Table 1. The PEC formation occurs when protonated amine group of CH interacts electrostatically with the negatively charged carboxylic group of OKG as depicted in Figure 1.

No complexation was observed on addition of OKG solution to CH solution at ratio of 1 : 9. As the concentration of OKG increased there was a steady increase in yield till ratio of 7 : 3; after that the yield started to decrease suggesting that at this ratio maximum amount of OKG interacts with the CH and beyond that point increasing the amount of OKG will only result in solubilization of gum in the solution [10].

### 3.3. Viscosity Measurements

**Figure 2:**
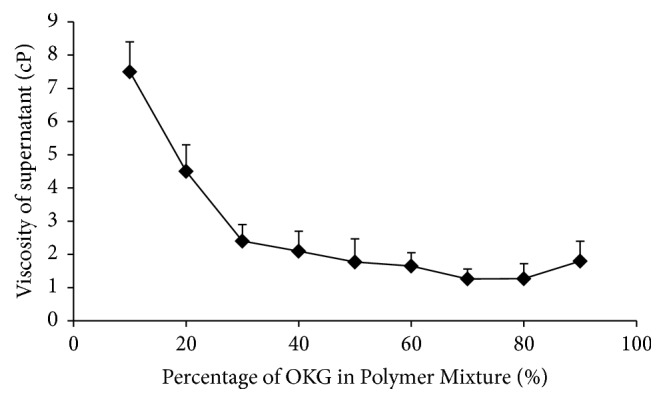
Effect of increasing the percentage of OKG in polymer mixture on viscosity of supernatant.

The graph shows the viscosity of the supernatant obtained after PECs were removed from the solution. OKG is anionic polymer due to presence of –COO^−^ groups while CH is cationic due to presence of NH_3_^+^ when ionized. A decline in the viscosity of the supernatant was observed as the concentration of OKG in the PECs increases (Figure 2).

This decrease in viscosity is observed till the concentration of 8 : 2 is reached. A further increase in OKG leads to slight increase in the viscosity. Previous studies have also reported a drop in viscosity to minimum when oppositely charged polymers like CH and carrageenan [26] or CH and chondroitin [27] are mixed. A slight increase in the viscosity of solution could be attributed to the presence of free OKG in the solution after the maximum interaction occurs as explained earlier by Chavasit and Torres [10].

### 3.4. FTIR Measurements

**Figure 3:**
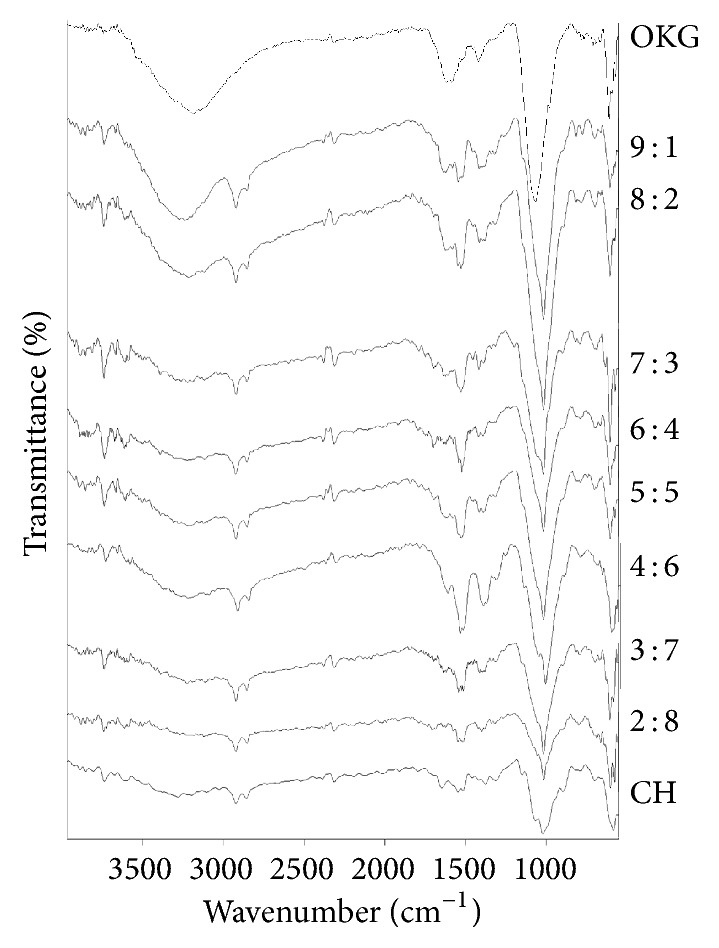
The FTIR spectra of OKG, PECs of ratios 2 : 8, 3 : 7, 4 : 6, 5 : 5, 6 : 4, 7 : 3, 8 : 2, and 9 : 1, and CH showing characteristic bands.

The FTIR spectra of OKG, CH, and PECs are depicted in Figure 3.

The presence of absorption band at 3180.14 cm^−1^ in the FTIR spectra of OKG represents the vibrational stretches due to hydroxyl groups participating in hydrogen bonding that correspond to the basic carbohydrate structure of the polysaccharides. The FTIR spectra of OKG show an absorption peak at 1585.05 cm^−1^ which indicates the presence of carboxylic acid group in the form of –COO^−^ corresponding to antisym stretch and at 1419.42 cm^−1^ due to sym stretch. The absorption spectra of CH show two distinct peaks at 3736.65 cm^−1^ and 3606.38 cm^−1^ which also correspond to the hydroxyl groups. Two characteristic peaks at 2922.56 cm^−1^ and 2857.65 cm^−1^ are observed in the FTIR spectra of CH which corresponds to –CH_3_ and –CH_2_– due to antisym and sym stretching. These peaks are not present in OKG but in the PECs these two peaks are very prominent [28]. As the concentration of OKG increases in the PECs from 2 : 8 to 9 : 1, the broad hydrogen bonding peak becomes more prominent but the single distinct peaks are also visible. As the PECs are formed it was noticed that the peak of carboxylate ion shifts to the peak of acid alone and appears in the range of 1690–1710 cm^−1^ which indicates that the –COO^−^ is getting converted to –COOH. This conversion is indicative of formation of electrostatic bond which requires the transfer of counterion from one polymer to another. CH FTIR spectra show characteristic peak of primary amine at 3410.05 cm^−1^ and 1646.94 cm^−1^. Both are distinctively visible in the PECs of all concentrations. FTIR spectra of both CH and OKG show sharp peaks in the region of 1015–1065 cm^−1^ which correspond to the cyclic alcohols which are present in both the polymers. These peaks are also seen in all the PECs [28].

### 3.5. Thermal Analysis

The DSC of the pure OKG and its complexes with CH is tabulated in Table 1.

The OKG polymer thermogram depicts a broad endothermic peak starting from 45°C and shouldering up to 105.2°C with peak maxima of 55.062°C which may be due to close endothermic changes of glass transition (*T*_g_) and water loss [29]. The peak that represents removal of water present in the bound state is higher than the boiling point of water as more amount of heat is required to break the ionic bonds that water has made with the polymer [30]. CH showed an endothermic peak at 80.22°C and an endothermic peak at 311.30°C. The DSC thermograms of PECs show endotherms ranging from peak maxima from 231.241°C to 243.356°C with varying amounts of heat transfer for different concentration ratios. These transitions could be associated with cleavage of electrostatic interactions between oppositely charged constituents of OKG and CH since it is not observed in pure components [31].

### 3.6. Zeta Potential Measurements

**Figure 4:**
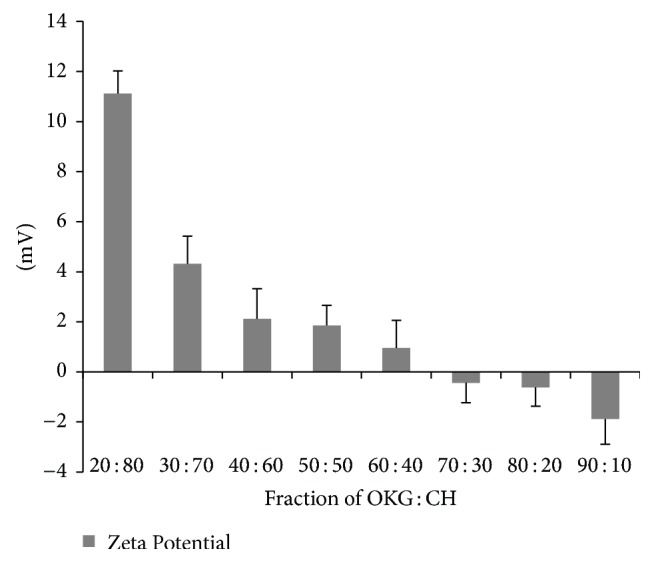
Effect of different ratios on zeta potential.

Zeta potential of pure CH is 12 mV and OKG is −11.47 mV which is due to presence of free amino groups in CH and free carboxylic groups of OKG giving it positive and negative net charge, respectively. The decrease in the zeta potential values was observed when the concentration of OKG increases in the PECs (Figure 4).

This decrease suggested the neutralization of the free positive charge associated with ionized CH upon the addition of OKG. The zeta value was reduced to −0.434 mV at the ratio of 7 : 3. This indicated a complete neutralization of all the free charges associated with CH and a maximum interaction. Zeta potential values drop further to negative values as after that the increase in OKG concentration only leads to increase in free negative charge that is associated with OKG [32].

### 3.7. Swelling Measurements

**Figure 5:**
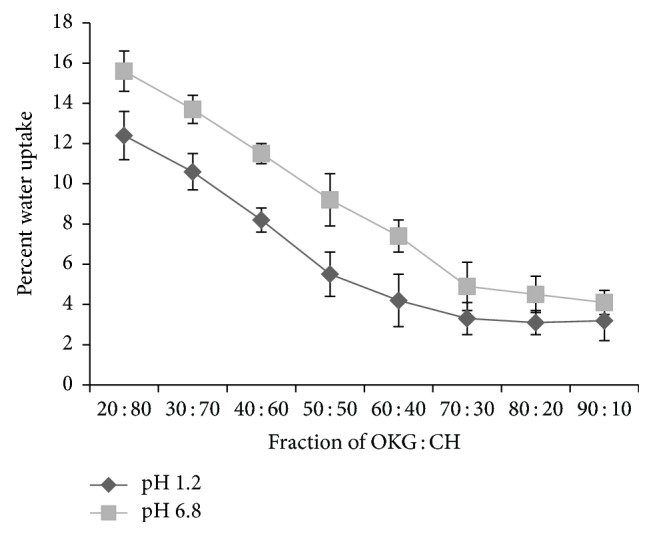
Effect of different ratios on percent water uptake by the PECs.

The water uptake capacity of the polymers was determined in pH 1.2 and phosphate buffer pH 6.8.  Figure 5 represents the water uptake as it is represented as percentage weight gain of total weight of the PECs. As the concentration of OKG increases in the PECs a decrease in the water uptake in both the pH media was observed till a ratio of 7 : 3 after which no significant change in the weight gain was observed. Uptake of water is associated with the ability of unionized groups present in polysaccharides to form ionic bonds with water. An increase in the concentration of OKG decreases the number of free ionic groups; as a result there is less water uptake. After 7 : 3 slight increase in the water uptake was observed. However, it is not significant and may be due to free ionic groups of OKG which may uptake the water molecules forming hydrogen bonding [24].

Another noteworthy observation is that percent water uptake of PECs at pH 1.2 is less as compared to pH 6.8. The reason for this observation may be the difference in the ionization of the two polymers at different pH. In acidic conditions the amine group of CH is protonated which causes electrostatic interactions of carboxylic group of OKG with protonated CH to produce a tight network which may lead to lower water uptake at lower pH. As the pH is higher the protonation of the CH decreases which leads to decrease in electrostatic interaction and hence the network of the matrix is loose which may lead to higher water uptake in the free space. This phenomenon was explained by Fahmy and Fouda in PECs of alginate and CH [33] and gum kondagogu and CH [34].

### 3.8. SEM Analysis

**Figure 6:**
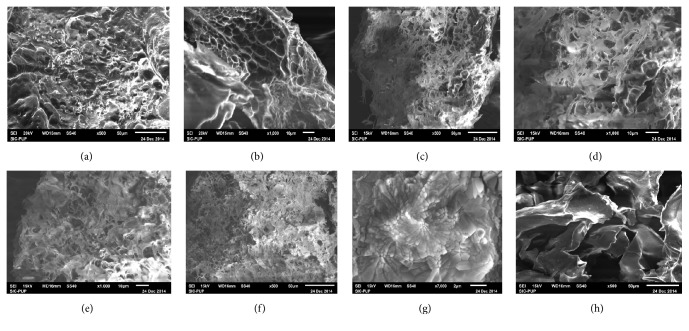
SEM images of different PECs between OKG and CH at concentration ratios 2 : 8 (a), 3 : 7 (b), 4 : 6 (c), 5 : 5 (d), 6 : 4 (e), 7 : 3 (f), 8 : 2 (g), and 9 : 1 (h).

The morphology of the PECs between OKG and CH at concentration ratios ranging from ratios 2 : 8 to 9 : 1 is shown in the Figure 6 as observed by SEM.

The PECs were dried using freeze drying which formed highly porous matrixes of the complexes as water from the surface sublimes damping the surface from which it sublimes. The cross linking between the oppositely charged polymers may also be responsible for the formation of sponge like matrix structure. The porosity of the PECs seems to decrease with an increase in OKG levels which is in consistence with water retention studies where structures with higher porosity in three-dimensional structures of the PECs tend to retain more water [23]. After the ratio where the maximum interaction is achieved, the additional OKG tends to reside at the interstitial surfaces of complex forming a smoother surface.

## 4. Conclusion

The PECs based on electrostatic interaction between OKG and CH were prepared by varying the ratios of two polymers using ionic gelation technique. The insoluble PECs obtained had a porous matrix and were characterized using FTIR and DSC studies. Further investigations suggest that the ratio of 7 : 3 leads to maximum interaction as implied by highest yield, lowest viscosity of supernatant, and lowest zeta potential at this ratio. Swelling studies show that swelling of PECs occurs more in pH 6.8 as compared to pH 1.2 which may in future be used in pH dependent sustained drug delivery system.

## Figures and Tables

**Table 1:** Effect of different ratios of OKG and CH on percentage yield and thermal properties of polyelectrolyte complexes.

OKG : CH	Percent yield (in %)	Thermal properties	
		Peak maximum (°C)	Heat (J/g)
1 : 9	0	-	-
2 : 8	36	231.241	122.989
3 : 7	40	237.622	88.628
4 : 6	44	243.356	110.485
5 : 5	54	239.529	63.955
6 : 4	58	242.384	84.341
7 : 3	60	242.042	60.377
8 : 2	54	240.543	84.087
9 : 1	20	239.475	68.245

## Supplementary Materials

Supplementary 1 SEM image of chitosan.

Supplementary 2 SEM image of Okra gum.
