# Supplementary figures and images for: Preparation and Characterization of Polyelectrolyte Complexes of Hibiscus esculentus (Okra) Gum and Chitosan
Source: Int J Biomater. 2018 Apr 24;2018:4856287. doi: 10.1155/2018/4856287 (PMC5941798; doi:10.1155/2018/4856287)

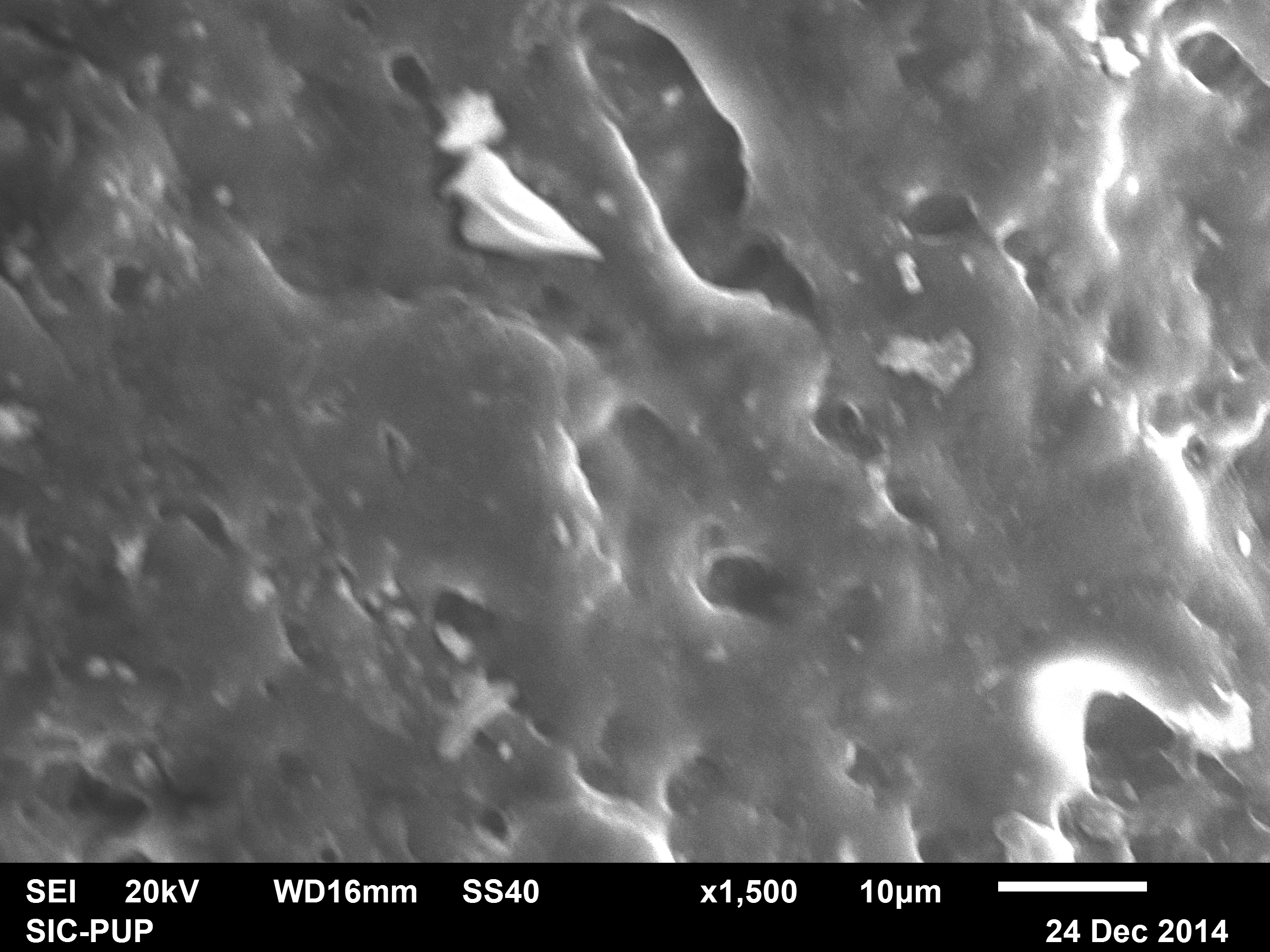

Supplement: Supplementary 1 — SEM image of chitosan. [file 4856287.f1.bmp]

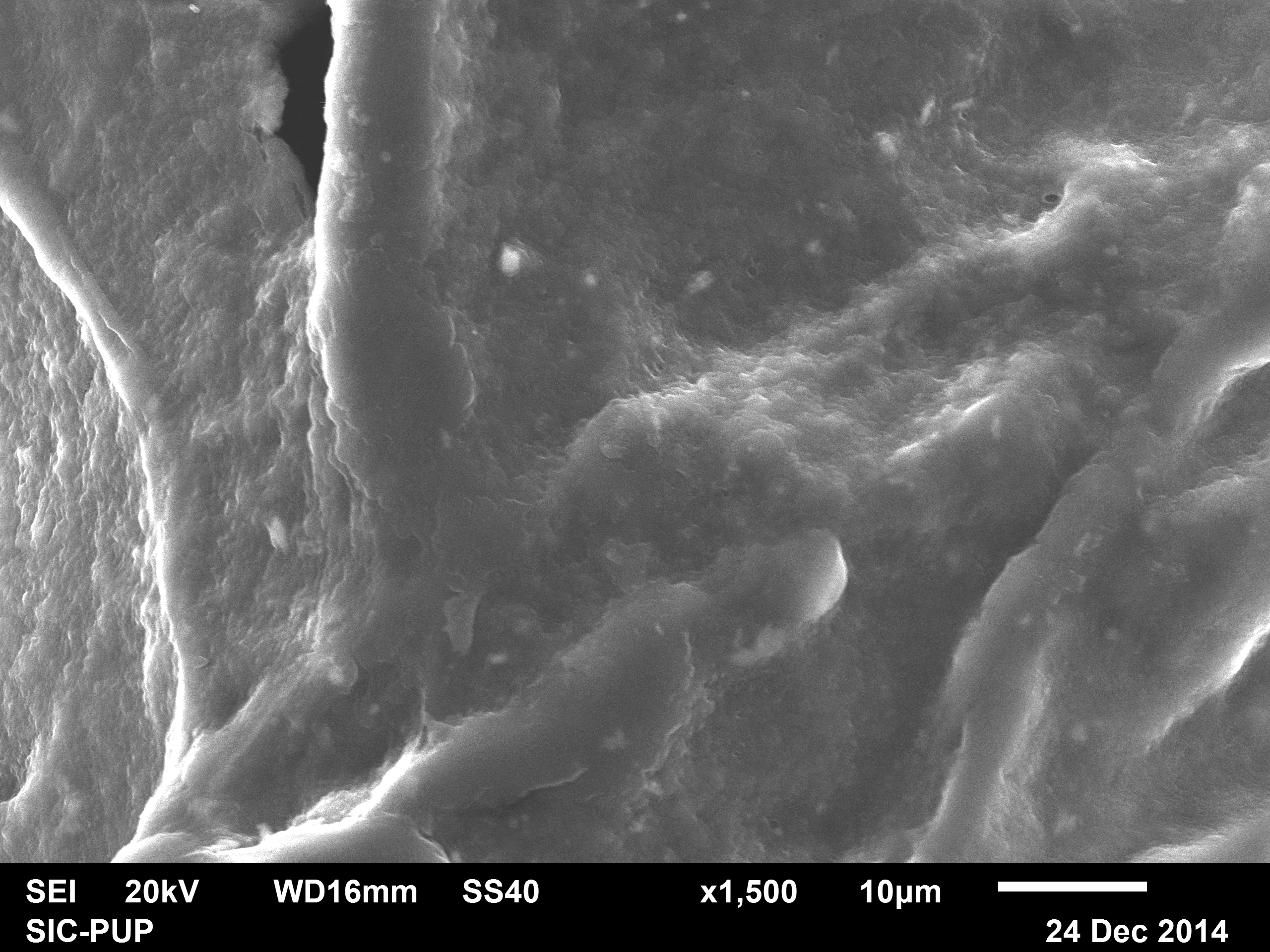

Supplement: Supplementary 2 — SEM image of Okra gum. [file 4856287.f2.bmp]
